# Cell cycle inhibitors improve seed storability after priming treatments

**DOI:** 10.1007/s10265-018-01084-5

**Published:** 2019-01-12

**Authors:** Naoto Sano, Mitsunori Seo

**Affiliations:** 1 RIKEN Center for Sustainable Resource Science, 1-7-22 Suehiro-cho, Tsurumi-ku, Yokohama, Kanagawa 230-0045 Japan; 2 Present Address: Institut Jean-Pierre Bourgin, INRA, AgroParisTech, CNRS, Université Paris-Saclay, 78000 Versailles, France

**Keywords:** Cell cycle, Chemical screening, Longevity, Priming, Seed desiccation, Seed germination

## Abstract

Seed priming is a treatment that controls seed water content to partially activate germination processes such as metabolism but prevents full germination of the seeds. The treatment is well known to enhance seed performance, including germination, but sometimes reduces seed storability or longevity as a side effect. Toward developing a novel priming technique that can maintain seed longevity for a longer time period, chemicals that suppress the seed deterioration under a controlled condition were screened from 80 known biologically active compounds contained in the RIKEN NPDepo authentic library using *Arabidopsis thaliana* seeds. Seeds primed with mimosine, a cell cycle inhibitor, retained higher survival rate after a controlled deterioration treatment compared to seeds primed without the chemical. In addition, other cell cycle inhibitors such as aphidicolin, hydroxyurea and oryzalin had similar effects on the seed storability after priming. Our results suggest that progression of the cell cycle during priming is an important checkpoint that determines the storability of seeds after the treatment.

**Electronic supplementary material:**

The online version of this article (10.1007/s10265-018-01084-5) contains supplementary material, which is available to authorized users.

## Introduction

Germination, often defined as the protrusion of part of the embryo from the surrounding endosperm and/or seed coat (testa) due to elongation of embryonic cells, is triggered by imbibition of dry seeds. After germination, the embryo develops into the seedling with green cotyledons. Seed stocks with a high rate of synchronized and rapid germination are required for industrial seedling production in nurseries (Macovei et al. 2017). Seed priming is a widely used commercial technique to improve seed performance, including germination uniformity, germination speed and stress tolerance of germinated seedlings. The treatment involves (1) imbibition of seeds under controlled conditions to trigger metabolic processes that are normally activated during early phases of germination, and (2) subsequent drying of the seeds prior to germination and loss of seed desiccation tolerance so that seeds reenter a quiescent state still viable (Dekkers et al. 2015; Paparella et al. 2015; Varierl et al. 2010). Several kinds of priming treatments have been developed so far (Paparella et al. 2015). Hydropriming is a simple and the most traditional type of priming during which seeds are soaked in water under an optimal temperature (usually in the range from 5 to 20 °C depending on species and cultivars) for a defined period. Imbibition at a suboptimal (high or low) temperature before sowing also promotes germination depending on the plant species and is referred to as thermopriming. However, non-controlled water up-take by seeds with hydropriming or thermopriming sometimes causes uneven hydration, which results in non-synchronous germination (McDonald 2000). In contrast to hydropriming and thermopriming that do not limit water uptake into seeds, osmopriming, also a widely used technique, controls the seed water content in the presence of osmotica such as polyethylene glycol or inorganic salts of sodium, potassium and magnesium (most commonly NaCl, NaNO_3_, MnSO_4_, MgCl_2_, K_3_PO_4_ and KNO_3_). The effects of priming involve several processes, including repair of damaged molecules (e.g. DNA, RNA and proteins), activation of primary metabolism, progression of the cell cycle, and responses to oxidative stress (Macovei et al. 2017; Varierl et al. 2010; Wojtyla et al. 2016). However, priming technology still largely depends on empirical rules (Paparella et al. 2015); thus, in-depth knowledge of the molecular bases of seed priming is required to develop innovative new-generation tools especially for the seed industry (Macovei et al. 2017).

Aside from the benefits of priming to enhance seed germination, the priming treatments sometimes reduce seed longevity or storability (the total time span during which seeds remain viable) (Liu et al. 1996). Primed seeds are in a more advanced physiological status in terms of the germination process compared with seeds that have not been primed and are, thus, more susceptible to deterioration (Varierl et al. 2010). The loss of longevity during priming is a disadvantage for commercial distribution of high-quality seeds. Transcriptome analysis with bulked recombinant inbred lines derived from a cross between two accessions of *Arabidopsis thaliana* (L.) Heynh. revealed that higher levels of expression for genes related to brassinosteroid (BR) biosynthesis/signaling and cell wall modification were associated with shorter seed longevity (Sano et al. 2017). The same study also showed that seeds primed with a BR biosynthesis inhibitor, brassinazole (Brz), had prolonged longevities compared to seeds primed without the chemical, indicating that BR promotes the loss of seed longevity during priming.

Treatments of biological organisms with small molecules to specifically perturb cellular functions is commonly referred to as chemical biology (Dejonghe and Russinova 2017). As in the case of Brz, small molecules that regulate pre-germinative processes have potential uses in new priming techniques to improve primed seed longevity. Moreover, studies on the mode of action of these chemicals should identify the biological pathways involved in the loss of seed longevity during priming. In this study, we screened for chemicals that prevented the seed deterioration after priming using the model plant, *Arabidopsis thaliana*. By this approach, we identified mimosine, a known cell cycle inhibitor, as a compound that successfully enhanced seed viability. The relationship between the cell cycle and the seed deterioration after priming was further examined by testing three additional compounds whose mode of action interferes with the cell cycle. Our results suggest that the use of cell cycle inhibitors may offer an improved method of seed priming.

## Materials and methods

### Plant materials and growth conditions

The *Arabidopsisthaliana* natural accessions, Col-0 and Est-1 (CS39289), used in this study were obtained from the Arabidopsis Biological Resource Center (http://abrc.osu.edu/). Seeds used for the experiments were propagated as described previously (Sano et al. 2017).

### Physiological analyses

**Fig. 1:**
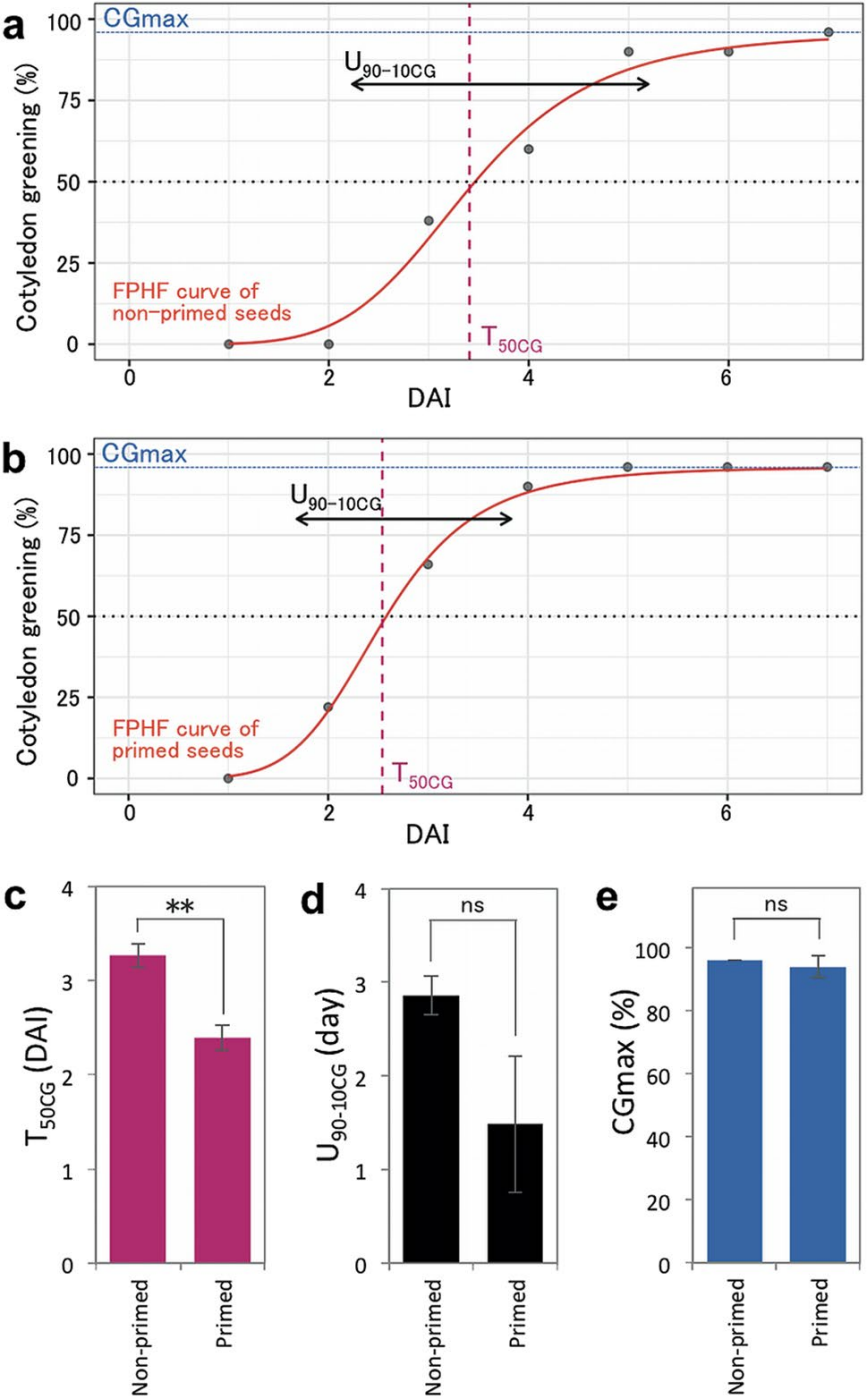
Effects of seed priming on parameters of seedling establishment. Growth curves for non-primed seeds (**a**) and primed seeds (**b**) fitted by the four-parameter hill function (FPHF). **c** Cotyledon greening speed (T_50CG_; time required for 50% of viable seeds to develop green cotyledons), **d** uniformity (U_90-10CG_; time interval between the 10 and 90% of viable seeds to develop green cotyledons) and **e** maximum value of cotyledon greening rate (CG_max_) of non-primed seeds and primed seeds (**P < 0.01, ns; not significant *P* > 0.05, Welch’s t-test)

**Fig. 2:**
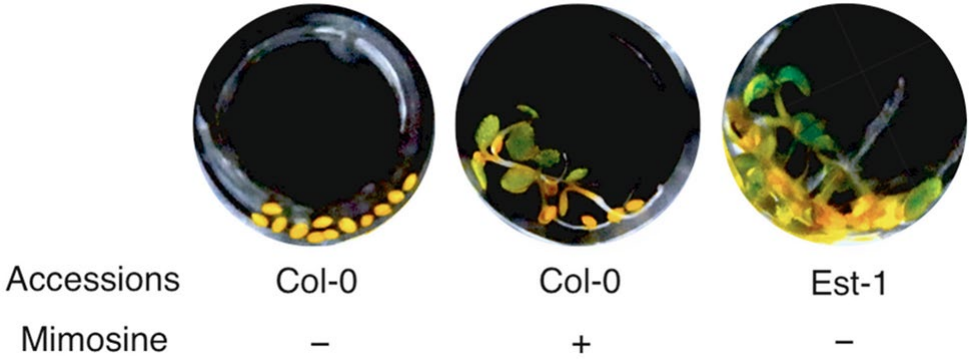
Identification of mimosine as a chemical that improves seed aging after priming. Col-0 seeds primed with (+) or without (−) mimosine were subjected to CDT for 3 days and then tested for their germination abilities. The concentration of mimosine was 34 µM. The Est-1 accession was used as a positive control that has higher resistance to CDT after priming. Photographs were taken 7 days after imbibition

**Fig. 3:**
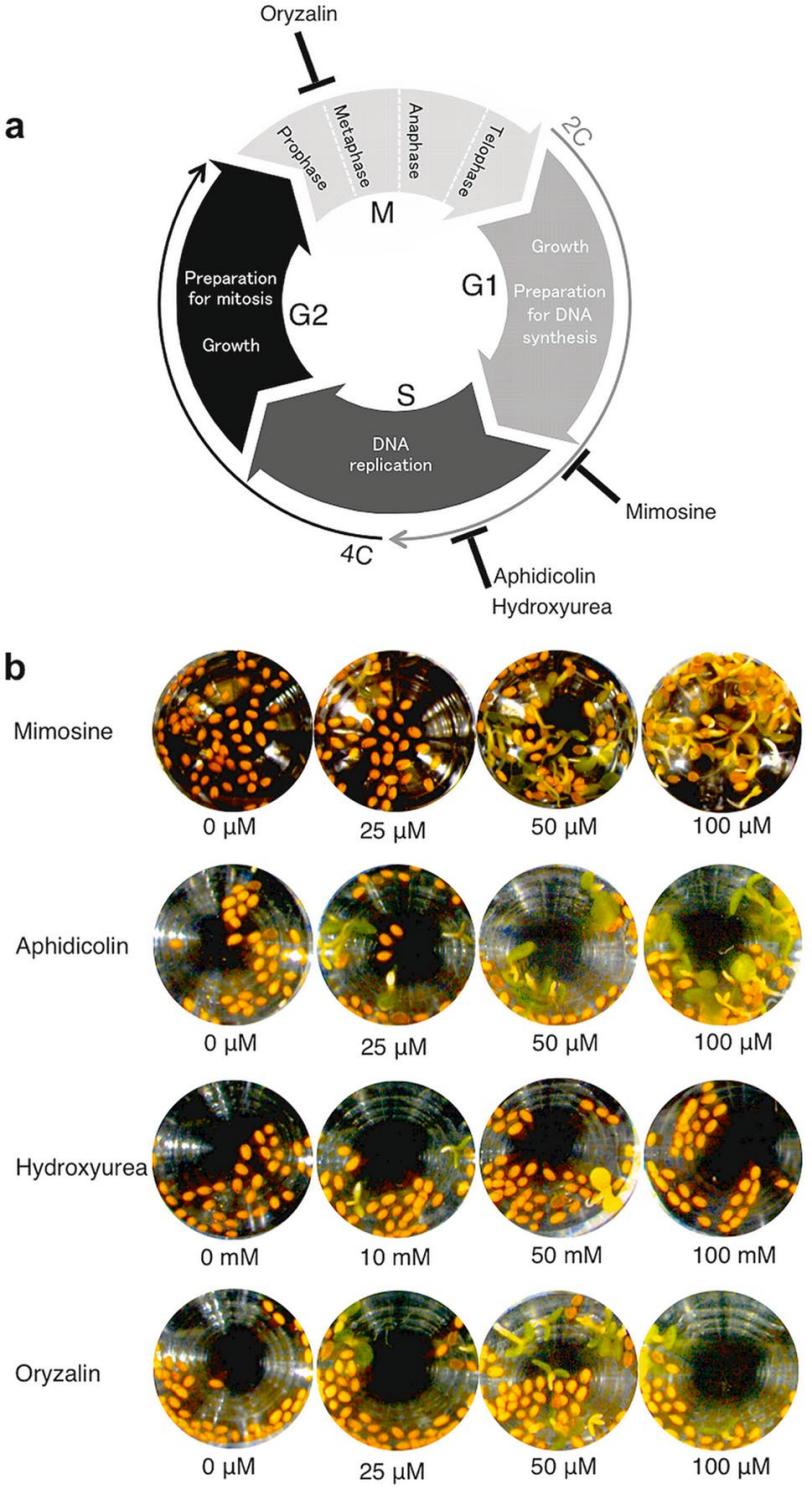
Effects of cell cycle inhibitors on seed aging after priming determined by a microplate assay. **a** A schematic diagram showing the cell cycle phases and target sites of mimosine, aphidicolin, hydroxyurea and oryzalin. G1: the period for cell growth during which nuclei have a 2C DNA content; S: the DNA replication phase that results in a doubling of the DNA content (from 2C to 4C); G2: the second growth period with nuclei having a 4C DNA content; and M: mitosis, during which genetic material is divided into two daughter nuclei (from 4C to 2C). **b** Col-0 seeds primed in the presence of various concentrations of cell cycle inhibitors were subjected to CDT for 3 days and then tested for their viabilities. Photographs were taken 7 days after imbibition

**Fig. 4:**
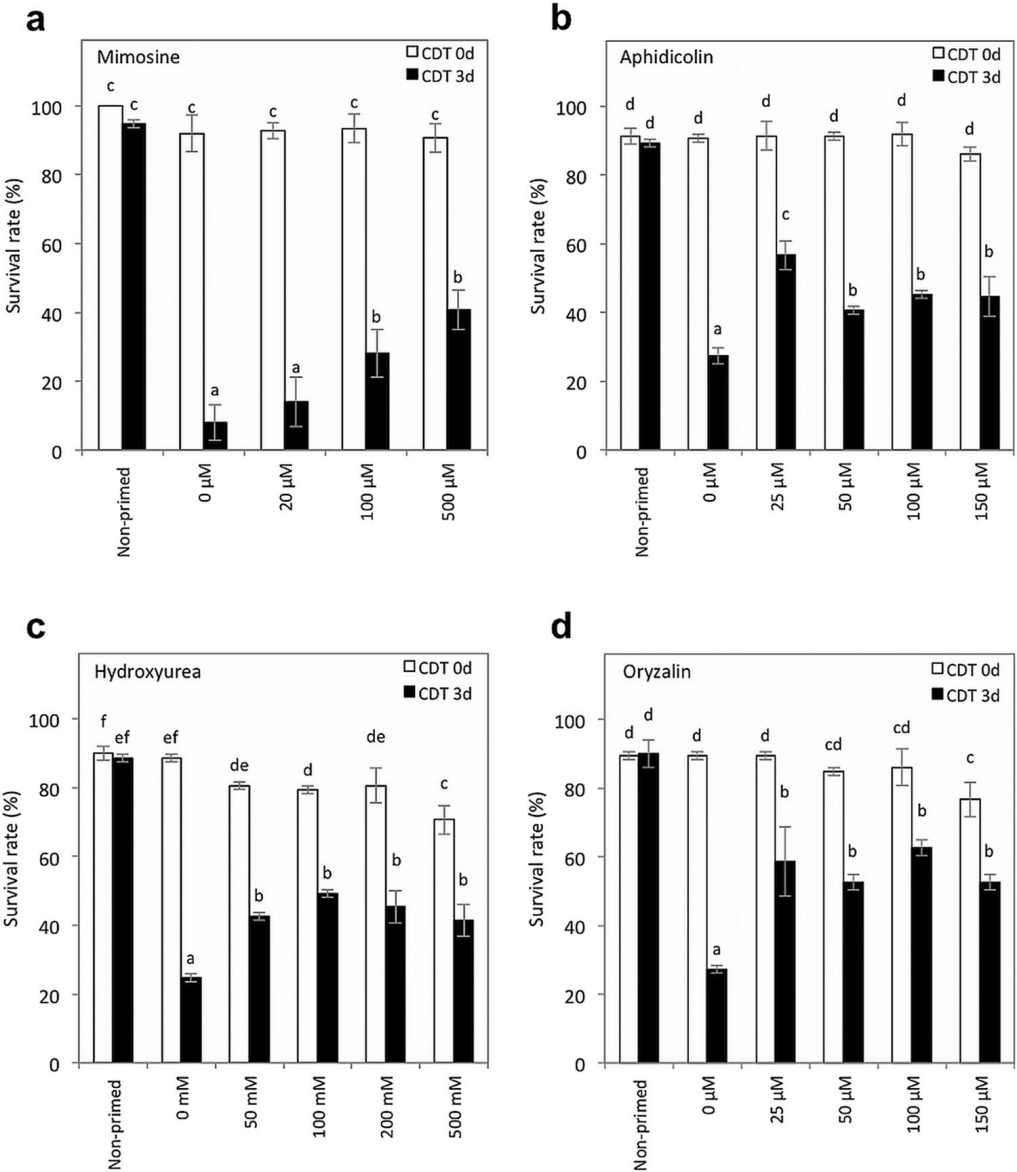
Quantitative determination of seed aging after priming with cell cycle inhibitors. Non-primed seeds and seeds primed with **a** mimosine, **b** aphidicolin, **c** hydroxyurea or **d** oryzalin were subjected to CDT for 0 or 3 days; survival rates of the seeds were scored 7 days after imbibition. Concentrations of chemicals used for the assays are indicated below the graphs. Values are means ± SD of three replicates. Different letters indicate significant differences (*P* < 0.05, Tukey–Kramer tests)

**Fig. 5:**
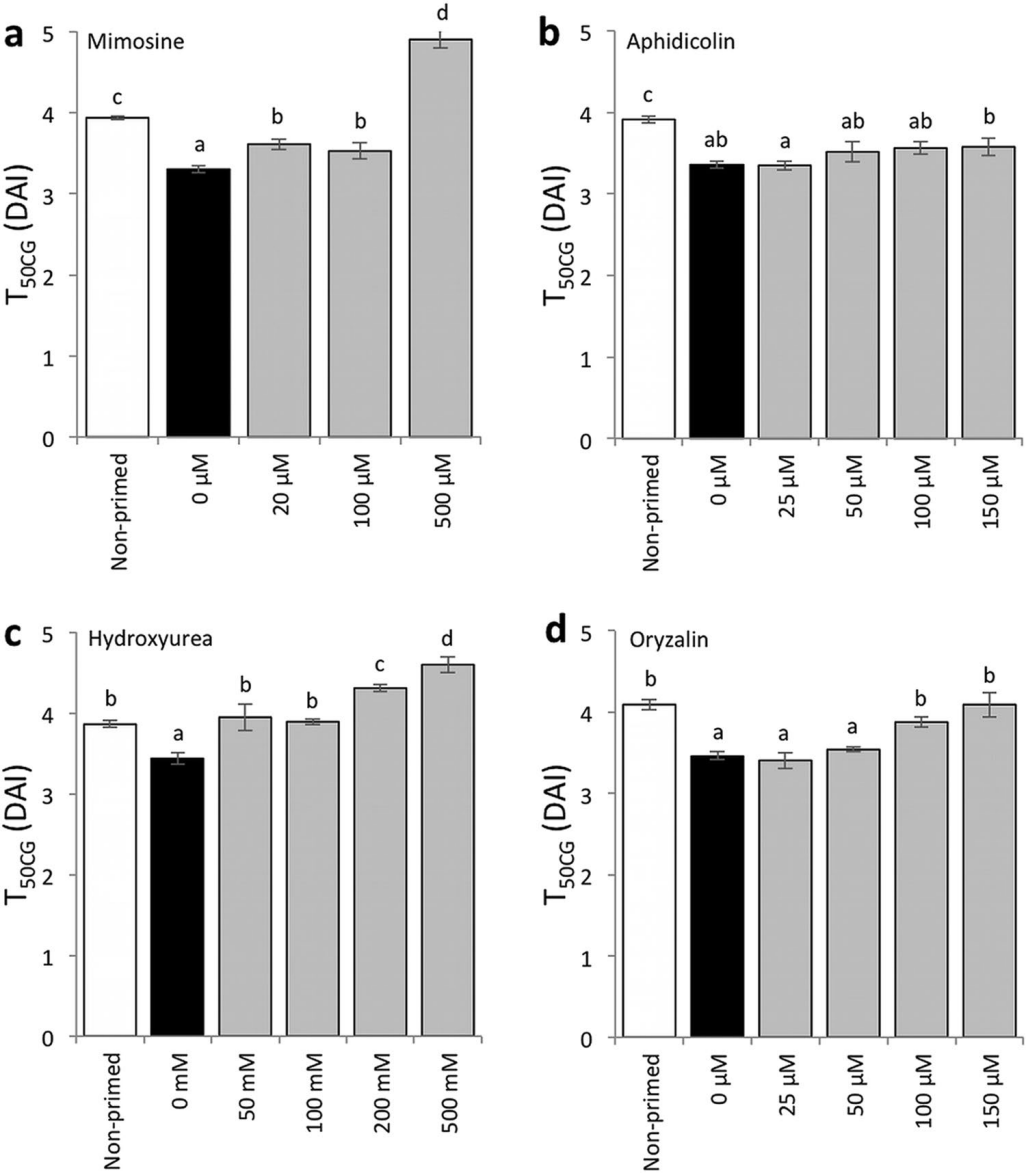
Effects of cell cycle inhibitors on cotyledon greening speed (T_50CG_) after priming. Greening speed of non-primed seeds and seeds primed with **a** mimosine, **b** aphidicolin, **c** hydroxyurea or **d** oryzalin were compared without CDT. Concentrations of chemicals used for the assays are indicated under the graphs. Values are means ± SD of three replicates. Different letters indicate significant differences (*P* < 0.05, Tukey–Kramer tests)

**Fig. 6:**
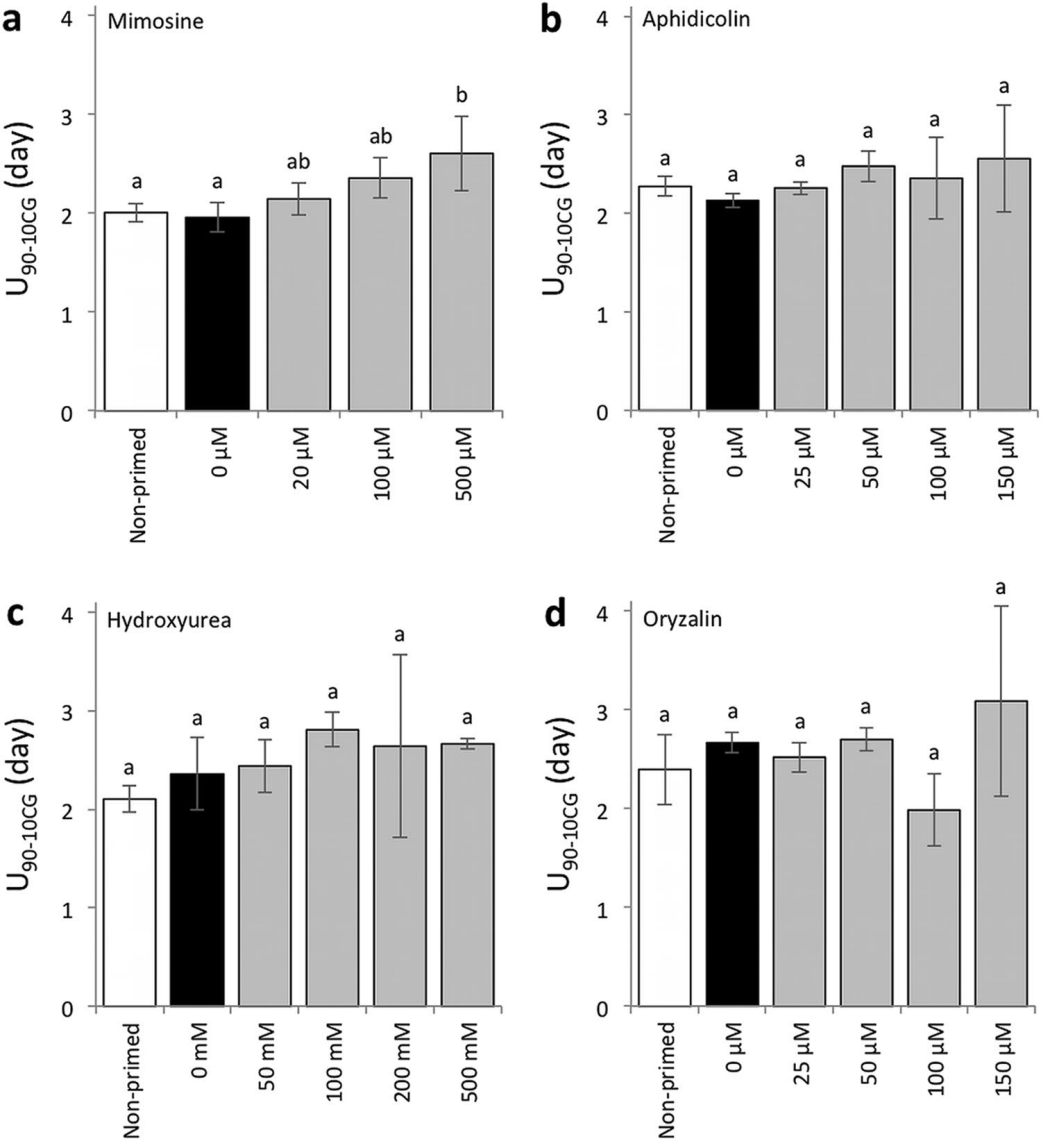
Effects of cell cycle inhibitors on uniformity of cotyledon greening (U_90-10CG_) after priming. Uniformity of non-primed seeds and seeds primed with **a** mimosine, **b** aphidicolin, **c** hydroxyurea or **d** oryzalin were compared without CDT. Concentrations of chemicals used for the assays are indicated under the graphs. Values are means ± SD of three replicates. Different letters indicate significant differences (*P* < 0.05, Tukey–Kramer tests)

**Fig. 7:**
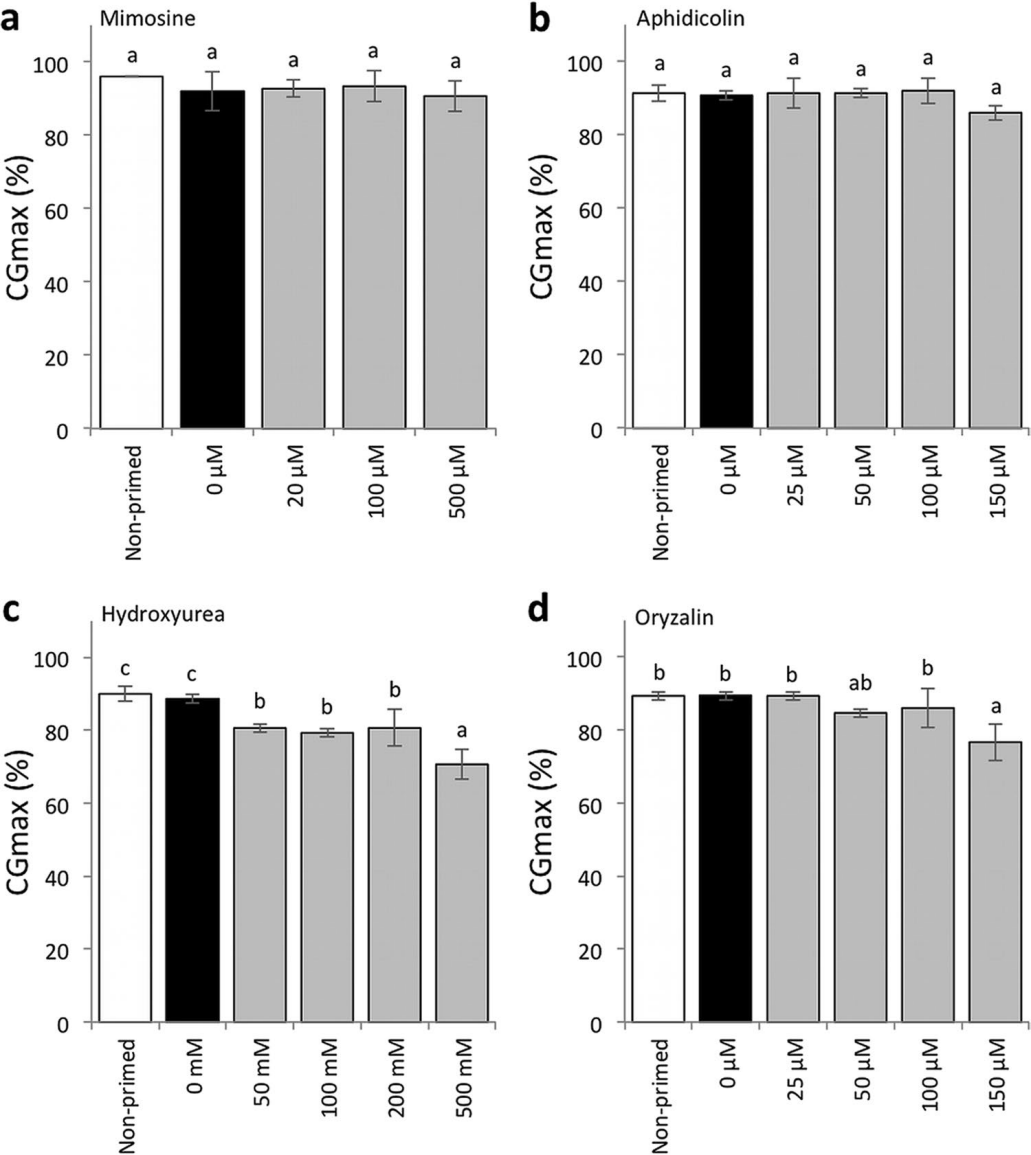
Effects of cell cycle inhibitors on maximum value of cotyledon greening rate (CG_max_) after priming. CG_max_ of non-primed seeds and seeds primed with **a** mimosine, **b** aphidicolin, **c** hydroxyurea or **d** oryzalin were compared without CDT. Concentrations of chemicals used for the assays are indicated under the graphs. Values are means ± SD of three replicates. Different letters indicate significant differences (*P* < 0.05, Tukey–Kramer tests)

For experiments presented in Figs. 1, 4, 5, 6, 7, S1, S2 and S4, seed priming was carried out as described previously (Sano et al. 2017). Briefly, surface-sterilized seeds in 1.5 ml tubes were imbibed at 4 °C for 3 days in the dark and then at 22 °C for 12 h under the light. Subsequently, the seeds were dried on filter papers under a laminar flow cabinet for 12 h, during which the seed weight became equivalent to that of non-primed seeds (Fig. S1). To estimate seed longevity, a controlled deterioration treatment (CDT) was performed by storing the seeds at a high temperature (37 °C) and high relative humidity (80%) condition as described previously (Sano et al. 2017). Natural seed aging was evaluated by storing the seeds in a moderate condition; 20 °C and 35% relative humidity. Germination assays were conducted using 0.8% (w/v) water agar plates with 50 seeds in triplicate. In the present study, seedling establishment and seed survival rates were scored based on the opening of green cotyledons following radicle emergence, since CDT often inhibits seedling establishment without affecting radicle emergence and healthy seedling establishment is an important trait associated with germination in agriculture. Seedling growth curve fitting and parameter analyses were performed using the package germinationmetrics version 0.1.1 (Aravind et al. 2018) in R version 3.3.2 (R Core Team 2016). The speed and uniformity of cotyledon greening (T_50CG_ and U_90-10CG_) were calculated based on T_50Germ_ and U_90-10_ in the package, respectively. For experiments presented in Figs. 2 and 3, seed priming and CDT were conducted using 96-well microplates with 30 seeds, and their viability was determined in distilled water on the same microplates in duplicate.

### Chemicals

The original RIKEN NPDepo authentic library contained 80 known biologically active compounds (http://www.cbrg.riken.jp/auth80/list.php) and they were solubilized in dimethylsulfoxide (DMSO) at a concentration of 10 mg ml^−1^. For the screening, original stock solutions were diluted 1:1500 with deionized water. l-Mimosine from *Koa hoale* seeds was purchased from Sigma-Aldrich and used for experiments presented in Figs. 3, 4, 5, 6 and 7. Aphidicolin, hydroxyurea and oryzalin were purchased from Wako Chemicals. Mimosine was directly dissolved in water containing 1% (v/v) DMSO at the concentrations used for the experiments. For other chemicals, stock solutions (100 × concentration) prepared in DMSO were added to water during priming. Water containing 1% (v/v) DMSO was used for control experiments.

### Statistical analyses

Welch’s t-tests were performed to determine significant differences of seedling establishment behavior (Fig. 1c–e) and seed weight (Fig. S1) before and after priming. Tukey–Kramer tests were used to determine significant differences in multiple comparisons of seedling establishment behavior and seed deterioration (Figs. 4, 5, 6, 7, S2).

## Results

### Chemical screening based on seed aging after priming treatment

We previously reported that *Arabidopsisthaliana* seeds dried after imbibition at 4 °C for 3 days in the dark and subsequent incubation at 22 °C for 12 h in continuous light conditions germinated faster than non-treated seeds when they were re-imbibed at 22 °C under continuous light (Sano et al. 2017). We confirmed that the weight of primed seeds was not significantly different from that of non-primed seeds (Fig. S1). In order to assess the effects of priming on seedling establishment behavior in more detail, the growth curves of non-primed and primed seeds were fitted using four-parameter hill function (FPHF) (El-Kassaby et al. 2008) and parameters of seedling establishment were estimated (Fig. 1a, b). We confirmed that our priming treatment remarkably reduced the time required for 50% of viable seeds to develop green cotyledons (T_50CG_; a parameter of seedling establishment speed) (Fig. 1c). On the other hand, the priming treatment did not significantly affect the uniformity (U_90-10CG_; time interval between the 10 and 90% of viable seed green cotyledons) although it appeared that the treatment slightly reduced the U_90-10CG_ value (Fig. 1d). This is possibly because unequal degree of seed hydration within the seed population, as our priming condition did not control it. In addition, the maximum value of cotyledon greening rate (CG_max_) was not affected by the priming treatment (Fig. 1e). We also reported previously that this priming treatment reduced seed storability, determined as the ability of seeds to establish healthy seedlings with green cotyledons after a CDT (Sano et al. 2017). To determine whether CDT is predictive for natural aging, we evaluated germination abilities of primed and non-primed seeds after storing the seeds in both the artificial condition (CDT at 80% relative humidity at 37 °C) and a more moderate room condition (35% relative humidity at 20 °C). Primed seeds of the Est-1 accession, previously shown to be more tolerant to CDT than Col-0 (Sano et al. 2017), showed significantly higher survival rates not only after CDT but also after aging at the room condition (Fig. S2), suggesting that CDT can be used to predict longevity of primed seeds under natural conditions.

To screen for chemicals that can improve seed aging after priming, we modified the assay to be conducted in 96-well microplates (Fig. 2). The cotyledons of Col-0 seeds primed without chemicals and subjected to a CDT for 3 days in the microplates did not green upon imbibition, whereas about 30% of the primed seeds survived after 3 days of a CDT in the experimental conditions as those presented in Fig. S2. It is likely that the effects of priming and/or a CDT on seed aging are slightly different between the two assays. Nevertheless, seeds of the Est-1 accession did not completely lose the ability to germinate and establish green cotyledons in the microplates (Fig. 2). Thus, we expected that chemicals that prevent seed deterioration after priming could be identified in this microplate assay. We used the RIKEN NPDepo authentic library, which is composed of 80 previously characterized and biologically active compounds, for the screening and found that seeds primed with ‘l-mimosine from *Koa hoale* seeds’ retained the ability to develop green cotyledons upon imbibition (Fig. 2).

### Effects of cell cycle inhibitors on seed aging after the priming treatment

Mimosine, β-(3-hydroxy-4-pyridon-1-yl)-l-alanine, is a non-protein amino acid that is produced in some tropical species of *Leucaena* and *Mimosa* (Selmar 1999). This chemical has allelopathic effects and is an inhibitor of cell division, including the capacity to block the cell cycle before the G1/S transition (Farinelli and Greene 1996; Vestena et al. 2001) (Fig. 3a). The effects of mimosine on the aging of primed seeds were tested at several concentrations in the microplate assay (Fig. 3b). Upon imbibition, some seeds developed into seedlings with green cotyledons when mimosine was present at concentrations higher than 50 µM during priming. However, it is possible that the recovery of cotyledon greening by mimosine might not be due to improved seed aging through the blockage of cell cycle, because the mode of action of the chemical in plant cells is not fully understood. Thus, we then determined whether other cell cycle inhibitors, such as aphidicolin (Ikegami et al. 1978), hydroxyurea (Young and Hodas 1964) and oryzalin (Morejohn et al. 1987), had similar effects on seed storability after priming. Concentrations of the chemicals used in the assays were established based on their inhibitory effects on the cell cycles as reported previously (Planchais et al. 2000). All three chemicals enabled some seeds to germinate and develop green cotyledons after priming and 3 days of CDT, although hydroxyurea was less effective than other compounds (Fig. 3b).

To statistically evaluate the effects of the cell cycle inhibitors, the priming treatment and CDT were performed as described previously (Sano et al. 2017) and germination assays were conducted using 0.8% water agar plates as were used in the experiments whose results are shown in Fig. 1 (Figs. 4, S3). Mimosine significantly improved the survival rate after CDT when the chemical was present at a concentration higher than 100 µM during priming (Fig. 4a). Similarly, when aphidicolin and oryzalin were present at concentrations higher than 25 µM during priming, the survival rate after CDT was reduced less than when primed without the inhibitors (Fig. 4b, d). Priming with hydroxyurea also improved the survival rate after CDT at concentrations higher than 50 mM (Fig. 4c).

### Effects of cell cycle inhibitors on seedling establishment speed

We then determined whether the priming treatments with cell cycle inhibitors were still effective in enhancing the seedling establishment of the seeds without CDT, in addition to their effects on improving seed aging after priming (Fig. S4). The speed of cotyledon greening (T_50CG_) was compared between seeds that were primed with or without the inhibitors (Fig. 5). Mimosine reduced the effect of priming in terms of shortening the T_50CG_ values, however, seeds primed with 20 and 100 µM mimosine still developed green cotyledons significantly faster than non-primed seeds (Fig. 5a). At all the concentrations tested, aphidicolin kept the effect of priming regarding the enhancement of cotyledon greening speed (Fig. 5b). Oryzalin also reduced the greening speed after priming, however, treatment with 25 and 50 µM oryzalin still had positive effects on cotyledon greening if compared with non-primed seeds (Fig. 5d). On the other hand, at any concentration tested, seeds primed with hydroxyurea did not green faster than seeds primed without the compound (Fig. 5c). We also analyzed the effects the cell cycle inhibitors on the uniformity of cotyledon greening (U_90-10CG_) and the maximum value of cotyledon greening rate (CG_max_) (Figs. 6, 7). The four cell cycle inhibitors did not affect the uniformity after priming at the concentrations tested expect that mimosine had a negative effect on the parameter when present at 500 µM during priming (Fig. 6a). The CG_max_ values were not changed by priming with mimosine or aphidicolin (Fig. 7a, b). On the other hand, hydroxyurea significantly reduced the values at all concentrations tested (Fig. 7c). Priming with 150 µM oryzalin also reduced CG_max_, while 25, 50 and 100 µM oryzalin did not affect the values (Fig. 7d).

## Discussion

In this study, we discovered that a cell cycle inhibitor, mimosine, prevented the seed deterioration after a priming treatment (Fig. 2). In addition, seeds that were primed with other cell cycle inhibitors such as aphidicolin, hydroxyurea and oryzalin also showed higher resistance to CDT compared with seeds primed without the chemicals (Figs. 3b, 4), suggesting that progression of the cell cycle is an important checkpoint to determine whether the seeds lose or maintain seed storability (or longevity) during priming.

In many plant species, higher cell cycle activities are observed during the early phases of seed development and the activities gradually decrease toward the quiescent state during seed maturation (Sliwinska 2000, 2009). In *Arabidopsis thaliana*, cell cycle activities increase until the torpedo/walking stick stages after fertilization, and embryos gradually lose their cell cycle activities during the maturation stages (Raz et al. 2001). Mature *Arabidopsisthaliana* seeds are composed of cells with a 2C or 4C DNA content, with the proportion of 2C nuclei more than 90% (Sliwinska et al. 2009). Upon imbibition, DNA replication was not observed in cotyledons until the endosperm ruptures, whereas cells in the hypocotyl-radicle axis had a higher DNA content (60% 2C, 39% 4C, and 1% 8C) at the time of germination. The proportion of the 4C nuclei in cotyledons greatly increased to more than 40% when root hair growth was observed after germination. These findings suggest that activation of the cell cycle and DNA replication upon imbibition are not uniform within an embryo. During priming of tomato (*Solanum lycopersicum*) and pepper (*Capsicum annuum* L.) seeds, the S to G2 transition of the cell cycle was observed in the radicle meristematic region with an increased percentage of cells having 4C nuclei (Bino et al. 1992; Lanteri et al. 1994). Accordingly, DNA content was proposed to be an index representing the effectiveness of priming treatments in accelerating subsequent germination (Lanteri et al. 1994). In contrast, the increase in nuclear DNA content by priming was reported to vary depending on the seed lot even though the germination of all lots tested was enhanced by priming (Gurusinghe et al. 1999). Possibly the effect of priming does not always require an increase in the DNA content in all plant species.

In pepper, the sensitivity of primed seeds to a CDT was associated with the ability of the cells to enter the S phase of the cell cycle and replicate DNA during the priming treatment (Saracco et al. 1995). Similarly, in tomato seeds, a negative correlation was observed between germination performance after storage and the cellular DNA content in primed seeds (Liu et al. 1996), a result that is consistent with our observation that priming with cell cycle inhibitors prevented the seed deterioration after priming (Figs. 3b, 4). These results suggest that an improved seed survival rate after priming with cell cycle inhibitors is associated with better maintenance of DNA replication upon imbibition. We found that the reduction of seed survival rate after CDT well correlated with that after storage at a natural condition (Fig. S2). However, we cannot exclude the possibility that the cell cycle inhibitors had specific protective effects against damages caused by CDT independently from inhibition of cell cycle.

Since cell cycle inhibitors delayed seed germination (Masubelele et al. 2005; Reigosa and Malvido-Pazos 2007), one may imagine that the chemicals also diminish the effect of priming to promote seed germination and subsequent seedling establishment. In fact, cell cycle inhibitors tested in our study reduced the cotyledon greening speed of primed seeds (Fig. 5); however, mimosine, aphidicolin and oryzalin did not completely suppress the effect of priming at specific concentrations. This suggest that optimization of chemical concentrations used for priming would enable to produce seeds with enhanced germination performance and acceptable longevity. In the present study, we adopted a simplified method for priming by using *Arabidopsisthaliana* as a model system. Thus, further studies will be required to determine whether the cell cycle inhibitors could be used to develop a new priming treatment for seeds of crop species in combination with already established commercial techniques.

## Electronic supplementary material

Below is the link to the electronic supplementary material.

Supplementary material 1 (PDF 2470 KB)

## Abbreviations

Brz: Brassinazole
BR: Brassinosteroid
CDT: Controlled deterioration treatment
DMSO: Dimethylsulfoxide

## References

[CR1] Aravind J, Vimala Devi S, Radhamani J, Jacob SR, Srinivasan K (2018) Germination metrics: seed germination indices and curve fitting. R package version 0.1.1. https://cran.r-project.org/package=germinationmetrics, 10.5281/zenodo.1219630. Accessed 20 Sept 2018

[CR2] Bino RJ, Vries JD, Kraak HL, Pijlen JV (1992). Flow cytometric determination of nuclear replication stages in tomato seeds during priming and germination. Ann Bot.

[CR3] Dejonghe W, Russinova E (2017). Plant chemical genetics: from phenotype-based screens to synthetic biology. Plant Physiol.

[CR4] Dekkers BJ, Costa MCD, Maia J, Bentsink L, Ligterink W, Hilhorst HW (2015). Acquisition and loss of desiccation tolerance in seeds: from experimental model to biological relevance. Planta.

[CR5] El-Kassaby YA, Moss I, Kolotelo D, Stoehr M (2008). Seed germination: mathematical representation and parameters extraction. For Sci.

[CR6] Farinelli SE, Greene LA (1996). Cell cycle blockers mimosine, ciclopirox, and deferoxamine prevent the death of PC12 cells and postmitotic sympathetic neurons after removal of trophic support. J Neurosci.

[CR7] Gurusinghe SH, Cheng Z, Bradford KJ (1999). Cell cycle activity during seed priming is not essential for germination advancement in tomato. J Exp Bot.

[CR8] Ikegami S, Taguchi T, Ohashi M, Oguro M, Nagano H, Mano Y (1978). Aphidicolin prevents mitotic cell division by interfering with the activity of DNA polymerase-alpha. Nature.

[CR9] Lanteri S, Saracco F, Kraak HL, Bino RJ (1994). The effect of priming on nuclear replication activity and germination of pepper (*Capsicum annuum* L.) and tomato (*Lycopersicon esculentum* L.) seeds. Seed Sci Res.

[CR10] Liu Y, Bino RJ, Van der Burg WJ, Groot SPC, Hilhorst HWM (1996). Effects of osmotic priming on dormancy and storability of tomato (*Lycopersicon esculentum* Mill.) seeds. Seed Sci Res.

[CR11] Macovei A, Pagano A, Leonetti P, Carbonera D, Balestrazzi A, Araújo SS (2017). Systems biology and genome-wide approaches to unveil the molecular players involved in the pre-germinative metabolism: implications on seed technology traits. Plant Cell Rep.

[CR12] Masubelele NH, Dewitte W, Menges M, Maughan S, Collins C, Huntley R, Nieuwland J, Scofield S, Murray JA (2005). D-type cyclins activate division in the root apex to promote seed germination in *Arabidopsis*. Proc Natl Acad Sci USA.

[CR13] McDonald MB, Black M, Bewley JD (2000). Seed priming. Seed technology and its biological basis.

[CR14] Morejohn LC, Bureau TE, Molè-Bajer J, Bajer AS, Fosket DE (1987). Oryzalin, a dinitroaniline herbicide, binds to plant tubulin and inhibits microtubule polymerization in vitro. Planta.

[CR15] Paparella S, Araújo SS, Rossi G, Wijayasinghe M, Carbonera D, Balestrazzi A (2015). Seed priming: state of the art and new perspectives. Plant Cell Rep.

[CR16] Planchais S, Glab N, Inzé D, Bergounioux C (2000). Chemical inhibitors: a tool for plant cell cycle studies. FEBS Lett.

[CR17] R Core Team (2016). R: a language and environment for statistical computing.

[CR18] Raz V, Bergervoet JHV, Koornneef M (2001). Sequential steps for developmental arrest in *Arabidopsis* seeds. Development.

[CR19] Reigosa MJ, Malvido-Pazos E (2007). Phytotoxic effects of 21 plant secondary metabolites on *Arabidopsis thaliana* germination and root growth. J Chem Ecol.

[CR20] Sano N, Kim JS, Onda Y, Nomura T, Mochida K, Okamoto M, Seo M (2017). RNA-Seq using bulked recombinant inbred line populations uncovers the importance of brassinosteroid for seed longevity after priming treatments. Sci Rep.

[CR21] Saracco F, Bino RJ, Bergervoet JHW, Lanteri S (1995). Influence of priming-induced nuclear replication activity on storability of pepper (*Capsicum annuum* L.) seed. Seed Sci Res.

[CR22] Selmar D, Wink M (1999). Biosynthesis of cyanogenic glycosides, glucosinolates and nonprotein amino acids. Biochemistry of plant secondary metabolism.

[CR23] Sliwinska E, Black M, Bradford KJ, Vazquez-Ramos J (2000). Analysis of the cell cycle in sugarbeet seed during development, maturation and germination. Seed biology: advances and applications.

[CR24] Sliwinska E (2009). Nuclear DNA replication and seed quality. Seed Sci Res.

[CR25] Sliwinska E, Bassel GW, Bewley JD (2009). Germination of *Arabidopsis thaliana* seeds is not completed as a result of elongation of the radicle but of the adjacent transition zone and lower hypocotyl. J Exp Bot.

[CR26] Varierl A, Vari AK, Dadlani M (2010). The subcellular basis of seed priming. Curr Sci.

[CR27] Vestena S, Fett-Neto AG, Duarte RC, Ferreira AG (2001). Regulation of mimosine accumulation in *Leucaena leucocephala* seedlings. Plant Sci.

[CR28] Wojtyla Ł, Lechowska K, Kubala S, Garnczarska M (2016). Molecular processes induced in primed seeds-increasing the potential to stabilize crop yields under drought conditions. J Plant Physiol.

[CR29] Young CW, Hodas S (1964). Hydroxyurea: inhibitory effect on DNA metabolism. Science.

