# Correction to: Cell cycle inhibitors improve seed storability after priming treatments

**DOI:** 10.1007/s10265-020-01194-z

**Published:** 2020-04-25

**Authors:** Naoto Sano, Mitsunori Seo

**Affiliations:** 1 RIKEN Center for Sustainable Resource Science, 1-7‑22 Suehiro‑cho, Tsurumi‑ku, Yokohama, Kanagawa 230-0045 Japan; 2 Present Address: Institut Jean-Pierre Bourgin, INRA, AgroParisTech, CNRS, Université Paris-Saclay, 78000 Versailles, France

## Correction to: Journal of Plant Research (2019) 132:263–271 10.1007/s10265-018-01084-5

The article Cell cycle inhibitors improve seed storability after priming treatments, written by Naoto Sano and Mitsunori Seo, was originally published Online First without Open Access. After publication in volume 132, issue 2, page 263–271 the author decided to opt for Open Choice and to make the article an Open Access publication. Therefore, the copyright of the article has been changed to © The Author(s) 2020 and the article is forthwith distributed under the terms of the Creative Commons Attribution 4.0 International License (https://creativecommons.org/licenses/by/4.0/), which permits use, duplication, adaptation, distribution and reproduction in any medium or format, as long as you give appropriate credit to the original author(s) and the source, provide a link to the Creative Commons license, and indicate if changes were made.

The original article was updated.

