# Supplementary material for: Cell cycle inhibitors improve seed storability after priming treatments
Source: J Plant Res. 2019 Jan 12;132(2):263–71. doi: 10.1007/s10265-018-01084-5 (PMC7205848; doi:10.1007/s10265-018-01084-5)
Supplement: Supplementary file 1 — Supplementary material 1 (PDF 2470 KB) [file 10265_2018_1084_MOESM1_ESM.pdf]

Electronic supplementary materials

Title:

Cell cycle inhibitors improve seed storability after priming treatments

Authors:

Naoto Sano and Mitsunori Seo

Journal:

Journal of Plant Research

Corresponding author:

Mitsunori Seo

RIKEN Center for Sustainable Resource Science, 1-7-22 Suehiro-cho, Tsurumi-ku, Yokohama, Kanagawa 230-0045, Japan

Tel: +81-45-503-9666

E-mail: mitsunori.seo@riken.jp

Content:

Figs. S1–S4

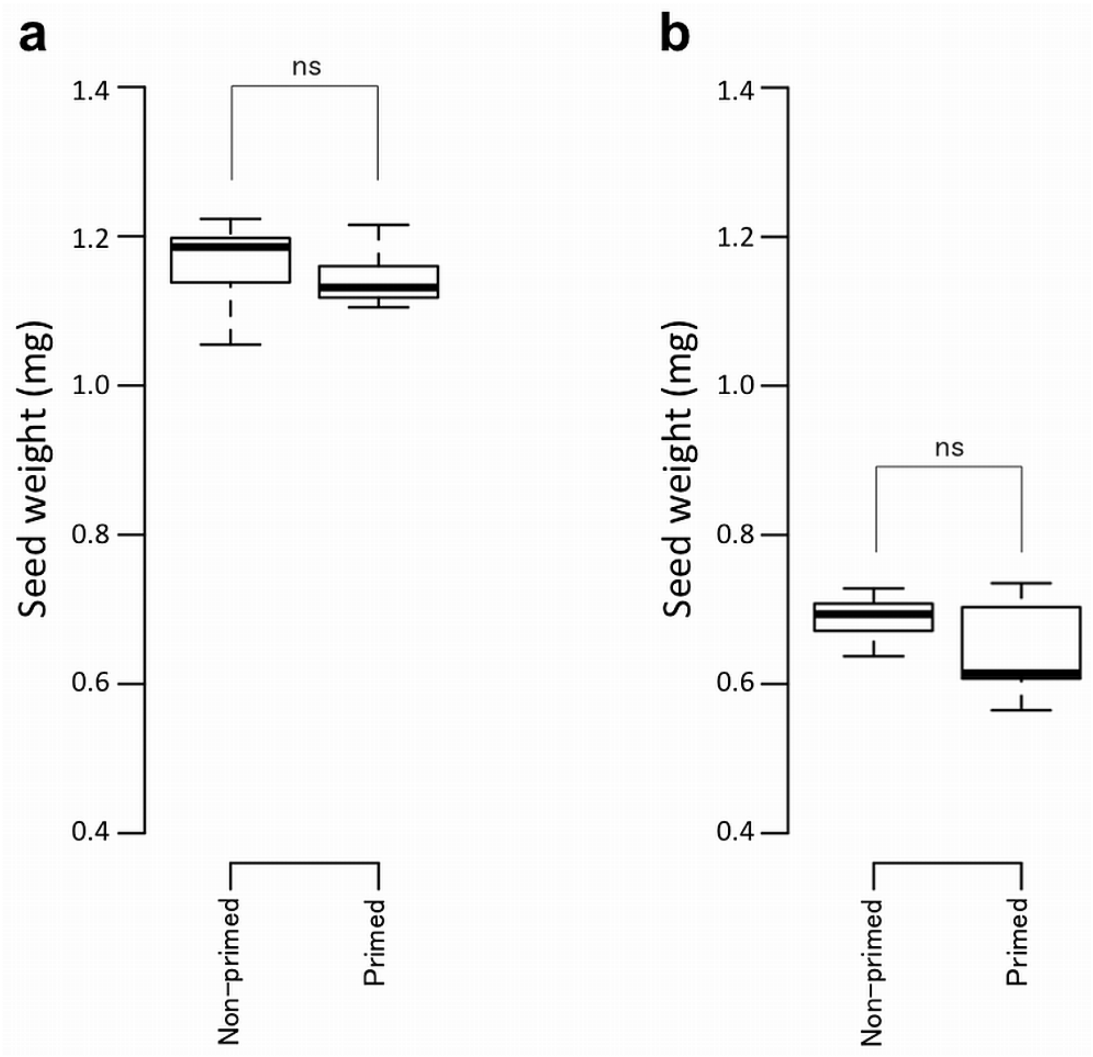

Fig. S1 Box plots of seed weight before and after priming. (a) Weight of 50 seeds before and after priming with 1.5 ml tubes. (b) Weight of 30 seeds before and after priming with 96-well microplates. Boxes delimit the first and third quartiles and the horizontal lines in the middle of the boxes represent median values. Whiskers delimit the lowest and the highest value within 1.5 of the interquartile range of the lower and the upper quartile, respectively (*n* = 9). ns, not significant (*P* > 0.05, Welch's t-test).

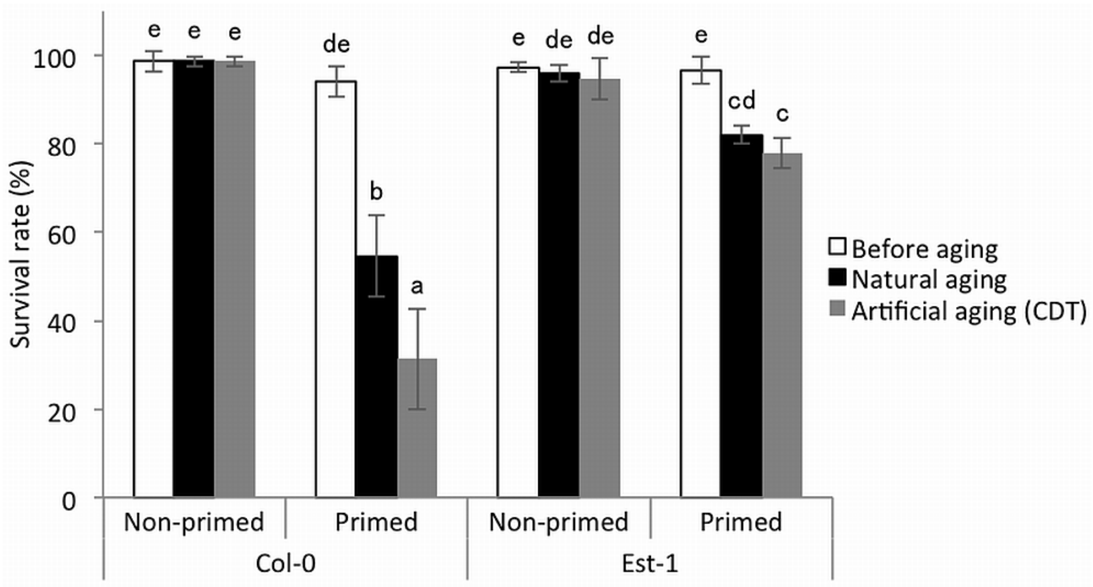

Fig. S2 Seed aging under a natural and an artificial seed storage condition. Primed and non-primed seeds of Col-0 and Est-1 accessions were stored at a natural condition (20°C, 35% relative humidity) for 3 months or an artificial aging condition (37°C and 80% relative humidity; CDT) for 3 days. Survival rates (or germination %) of the seeds were scored 7 days after imbibition. Values are means ± SD of three replicates. Different letters indicate significant differences ($P < 0.05$, Tukey-Kramer tests).

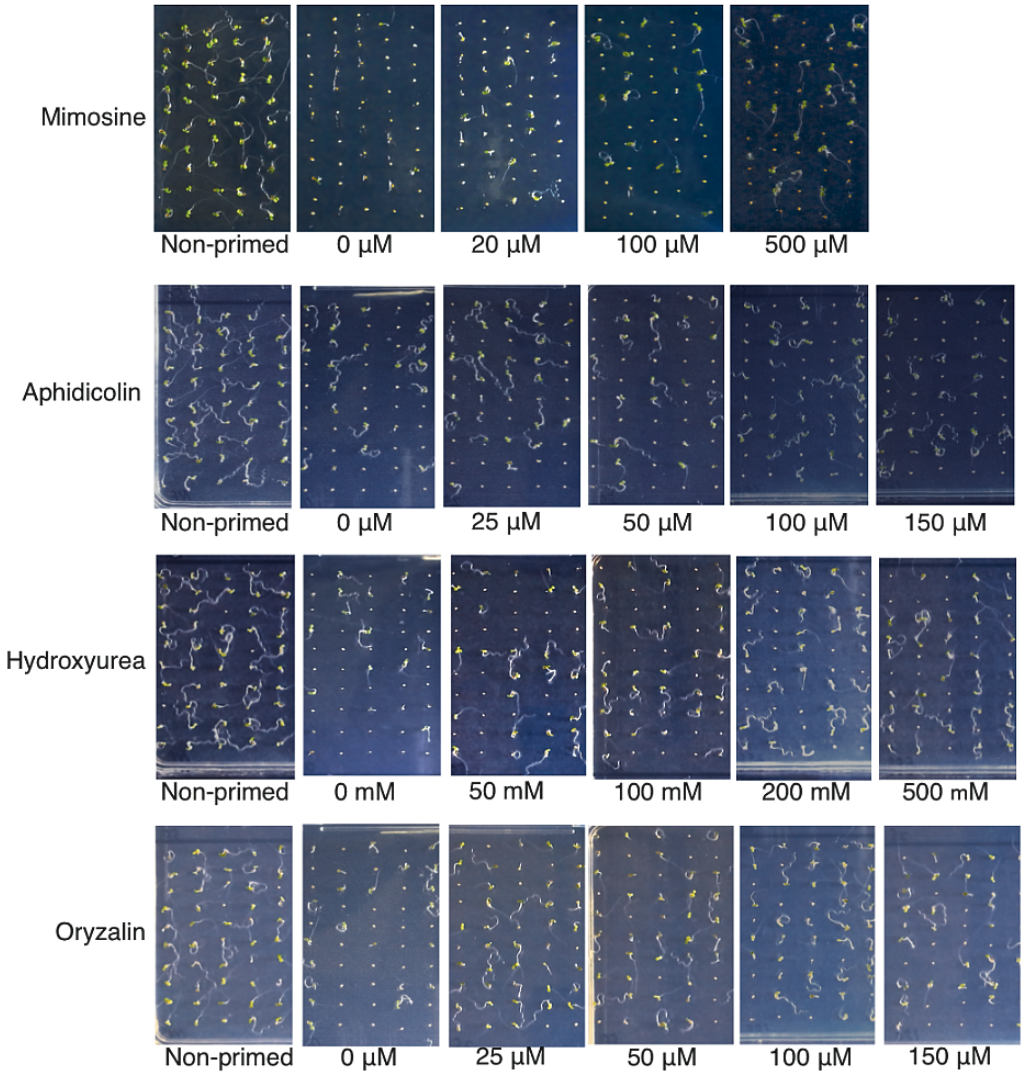

Fig. S3 Cotyledon greening abilities of non-primed seeds and seeds primed with cell cycle inhibitors after 3 days of a CDT. Photographs were taken 7 days after imbibition. Concentrations of chemicals used for the assays are indicated below the photographs.

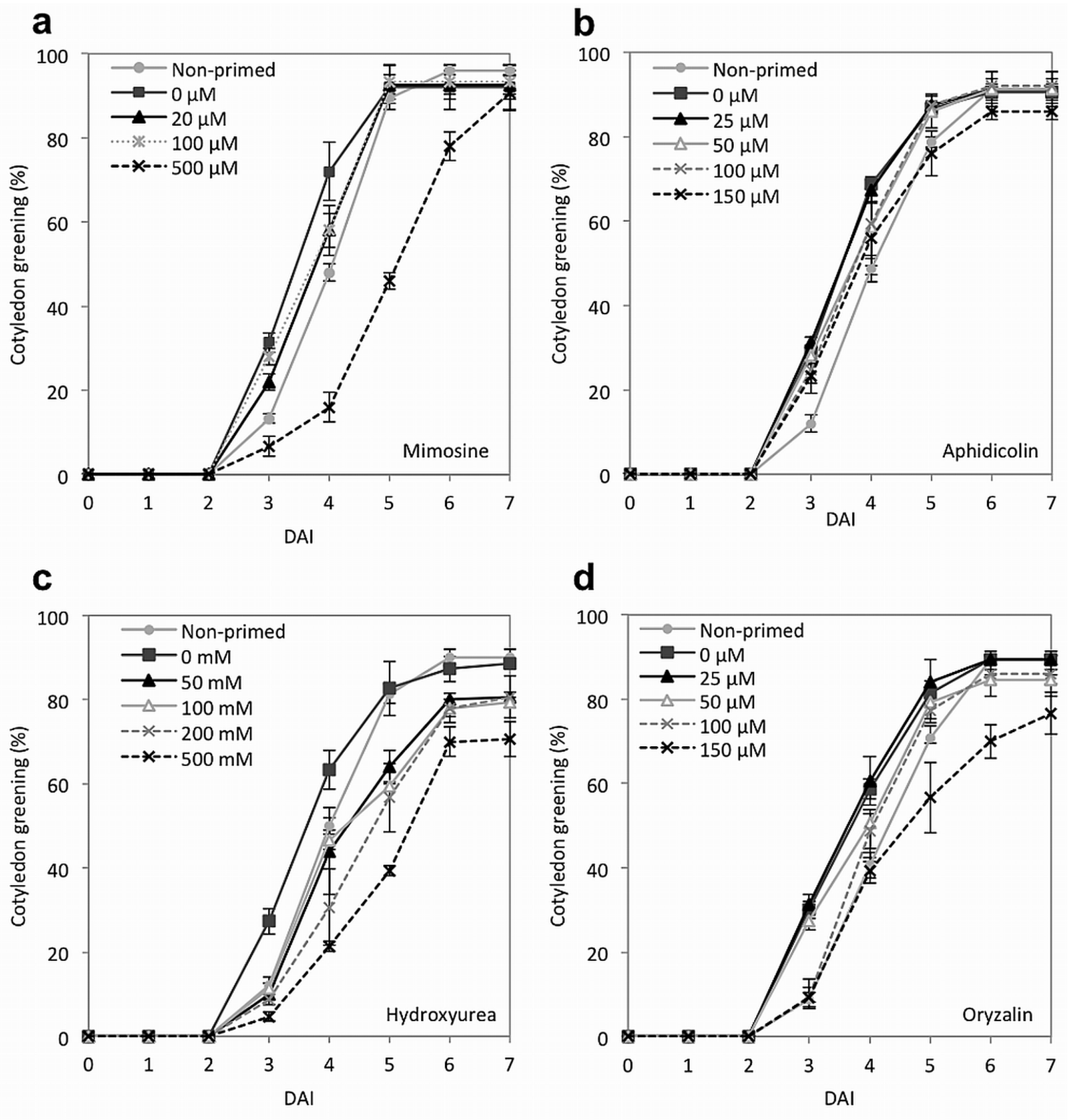

Fig. S4 Effects of cell cycle inhibitors on seedling establishment after priming. Cotyledon greening of non-primed seeds and seeds primed with (a) mimosine, (b) aphidicolin, (c) hydroxyurea or (d) oryzalin were compared without a CDT. Concentrations of chemicals used for the assays are indicated within each graph. Values are means ± SD of three replicates.
